# The phylogeography of *Fagus hayatae* (Fagaceae): genetic isolation among populations

**DOI:** 10.1002/ece3.2042

**Published:** 2016-03-21

**Authors:** Ling‐Xiao Ying, Ting‐Ting Zhang, Ching‐An Chiu, Tze‐Ying Chen, Shu‐Jin Luo, Xiao‐Yong Chen, Ze‐Hao Shen

**Affiliations:** ^1^ Department of EcologyCollege of Urban and Environmental SciencesThe MOE Key Laboratory of Earth Surface ProcessesPeking UniversityBeijing100871China; ^2^ School of Life SciencesPeking‐Tsinghua Center for Life SciencesPeking UniversityBeijing100871China; ^3^ Experimental Forest/Department of ForestryNational Chung Hsing University250 Kuokuang Rd.Taichung40227; ^4^ Department of Forestry and Natural ResourcesNational Ilan University 1Sec. 1Shen‐Lung RoadI‐Lan260; ^5^ College of Natural Resource and EnvironmentEast China Normal UniversityShanghai200062China

**Keywords:** cpDNA sequence fragments, *Fagus hayatae*, genetic isolation, nDNA SSR loci, phylogeography

## Abstract

The beech species *Fagus hayatae* is an important relict tree species in subtropical China, whose biogeographical patterns may reflect floral responses to climate change in this region during the Quaternary. Previous studies have revealed phylogeography for three of the four *Fagus* species in China, but study on *F. hayatae*, the most sparsely distributed of these species, is still lacking. Here, molecular methods based on eight simple sequence repeat (SSR) loci of nuclear DNA (nDNA) and three chloroplast DNA (cpDNA) sequences were applied for analyses of genetic diversity and structure in 375 samples from 14 *F. hayatae* populations across its whole range. Both nDNA and cpDNA indicated a high level of genetic diversity in this species. Significant fixation indexes and departures from the Hardy–Weinberg equilibrium, with a genetic differentiation parameter of *R*_st_ of 0.233, were detected in nDNA SSR loci among populations, especially those on Taiwan Island, indicating strong geographic partitioning. The populations were classified into two clusters, without a prominent signal of isolation‐by‐distance. For the 15 haplotypes detected in the cpDNA sequence fragments, there was a high genetic differentiation parameter (*G*_st_ = 0.712) among populations. A high *G*_st_ of 0.829 was also detected outside but not within the Sichuan Basin. Consistent with other *Fagus* species in China, no recent population expansion was detected from tests of neutrality and mismatch distribution analysis. Overall, genetic isolation with limited gene flow was prominent for this species and significant phylogeographic structures existed across its range except for those inside the Sichuan Basin. Our study suggested long‐term geographic isolation in *F. hayatae* with limited population admixture and the existence of multiple refugia in the mountainous regions of the Sichuan Basin and southeast China during the Quaternary. These results may provide useful information critical for the conservation of *F. hayatae* and other Chinese beech species.

## Introduction

*Fagus* L. had a widespread distribution across the continents of the Northern Hemisphere during the Tertiary (Denk et al. 2005; Denk and Grimm 2009), and the 10 species of *Fagus* are still among the dominant components in northern temperate forests (Shen 1992; Denk 2003). Unlike Europe and North America, where *Fagus* species dominate the climax forests in temperate regions (Forcier 1975; Peters 1997), the generally recognized four *Fagus* species endemic to China are restricted to the subtropical mountains, with a northern limit near 34°N (Cao et al. 1995; Fang and Lechowicz 2006). Among them, *Fagus hayatae* Palib. has the narrowest species range and is the only one disjunctly distributed from mainland subtropical mountains to Taiwan Island (Shen et al. 2015).

According to the contemporary patterns of genetic diversity and spatial population genetic structure, *Fagus* species were found to shrank southward with refugia distribution in the Quaternary Ice Ages in Europe (Demesure et al. 1996; Vettori et al. 2004; Vornam et al. 2004; Shanjani et al. 2010), North America (Kitamura and Kawano 2001), and Japan (Tomaru et al. 1998; Okaura and Harada 2002; Kobashi et al. 2006; Hiraoka and Tomaru 2009a), and then expanded northwards in the postglacial stage. The impacts of climate change on species distributions have also been revealed for *Fagus* species in China. Phylogeographical analysis of *Fagus engleriana* detected the effects of glacial periods and postglacial population dynamics (Lei et al. 2012), as also inferred by Zhang et al. (2013) from comparison of the demographic histories of *Fagus lucida* and *Fagus longipetiolata*.

*Fagus hayatae* is monoecious with unisexual flowers. Normally 4–6 male flowers constitute an umbrella capitulum and only 1–2 female flowers connect in a pedicel of *F. hayatae*. The cupule generally encloses two triangle‐shaped nuts with three shallow ridges. While *Fagus* species are wind‐pollinated (Faegri and van der Pijl 1979), Chen et al. (2011) suggested that *F. hayatae* suffered heavy seed dispersal limitation caused by predation of vertebrates, as also reported in other *Fagus* species (Ida et al. 2004). Unlike other three Chinese *Fagus* species with more extensive and coherent species ranges in subtropical mainland China, *F. hayatae* has a more restricted and fragmented species range, disjunctly distributed in a few remotely isolated high mountains in the north of subtropical China, and in the Taiwan Island (Hsieh 1989; Shen 1992; Li and Li 2008), and within an altitudinal range of 1000–2300 m (Zhang et al. 2013; Shen et al. 2015). Research on genetic diversity in *F. hayatae* was first performed by Kato et al. (2000), who detected no genetic diversity in mitochondrial DNA of 15 individuals. Kung (2012) analyzed the genetic structure of *F. hayatae* populations mainly from Taiwan (with three exceptions from continental China) using chloroplast and nuclear genome sequences, but the bias against analyses of the chloroplast genome weakened the phylogeographical analyses, and the demographic history of this species remains elusive.

In this study, we applied microsatellite markers of nuclear DNA (nDNA) and chloroplast DNA (cpDNA) sequence fragments to examine phylogeography of *F.  hayatae* in China and attempted to address the following three questions: (1) What is the spatial genetic structure in *F. hayatae* populations? (2) Where were the potential glacial refugia for *F. hayatae*? (3) What can be implied about the demographic history of *F. hayatae*? The results of this study should provide critical insights for understanding the distribution and history of Chinese beeches in the subtropical region, as well as the relationships among different *Fagus* species.

## Materials and Methods

### Population sampling and DNA extraction

**Figure 1:**
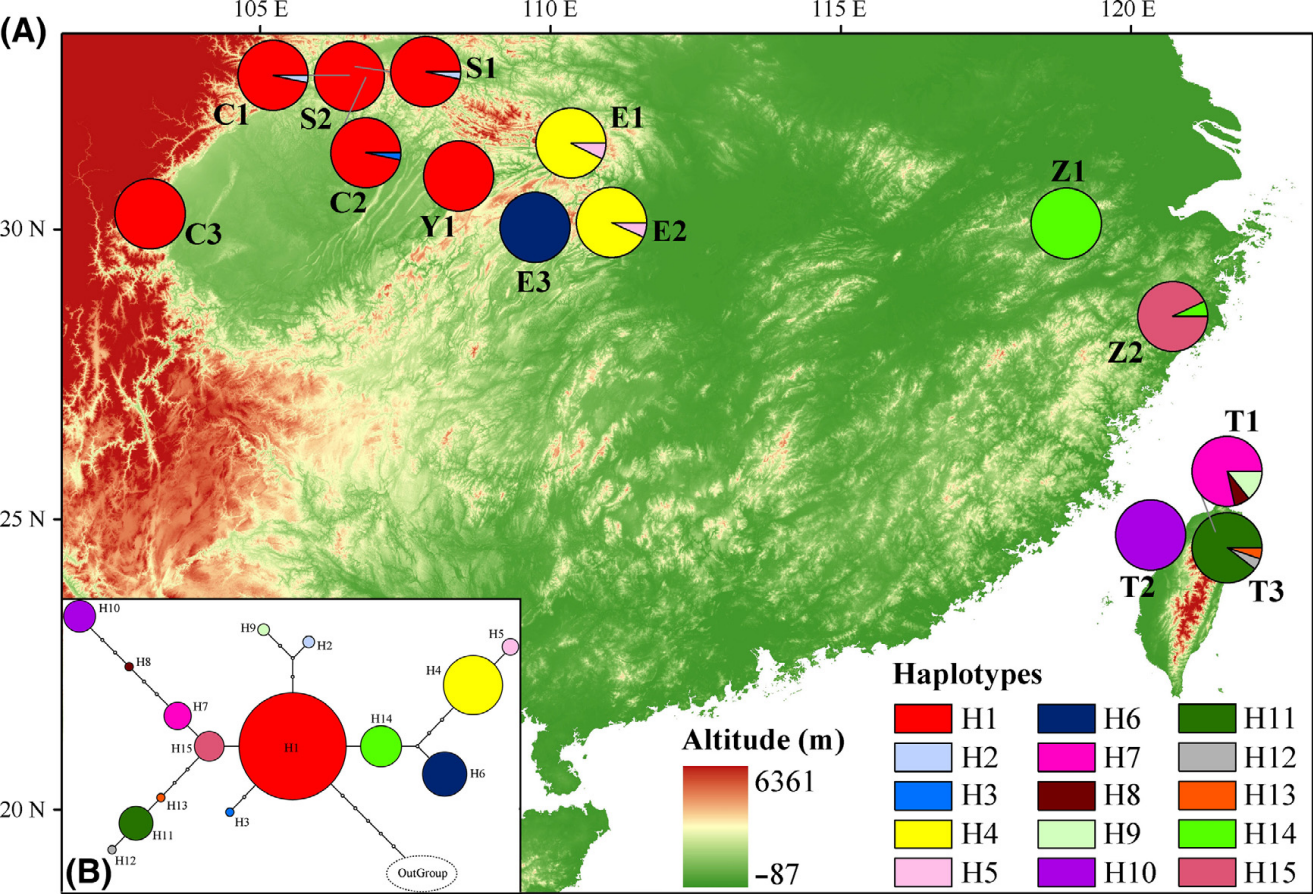
(A) Distribution of 15 haplotypes of *Fagus hayatae* and (B) their statistical parsimony network with “OutGroup” (*Fagus lucida*). The size of each colored circle is proportional to the relative haplotype frequency and the haplotype colors correspond to those shown in (A), while solid lines represent nucleotide site differences between haplotypes.

Field surveys and sampling of *F. hayatae* populations were implemented during July–September 2012, covering the whole distribution range of the species. A total of 375 individuals in 14 populations were sampled (Fig. 1A, Table 1). Young and healthy leaves were collected from each individual, and sampled individuals in each population were at least 30 m apart. All samples were dried with silica gel and stored at 4°C until needed.

**Table 1:** Sample locations, sizes, genetic diversity, and FI based on nDNA SSR loci in 14 populations of *Fagus hayatae*

Province (Abbreviation)	Code	Mountain	Lat. (N)	Long. (E)	Altitude (m)	Size	*A*	*H*_O_	*H*_E_	FI
Shaanxi (S)	S1	Liping	32°48′	106°38′	1317	30	9.75	0.621	0.692	0.229^a^
	S2	Micang	32°38′	106°32′	1794	30	8.38	0.565	0.642	0.029
Sichuan (C)	C1	Gucheng	32°39′	106°31′	1672	30	8.62	0.573	0.694	0.240^a^
	C2	Guangwu	32°37′	106°49′	1803	30	10.12	0.569	0.705	0.565^a^
	C3	Tiantai	30°16′	103°06′	1302	30	6.75	0.690	0.690	0.356^a^
Chongqing (Y)	Y1	Baizhi	30°55′	108°25′	1648	30	8.50	0.508	0.606	0.256^a^
Hubei (E)	E1	Shennongjia	31°29′	110°21′	1884	30	11.75	0.767	0.809	0.446^a^
	E2	Xiaozhaizi	30°07′	110°07′	1591	30	9.75	0.696	0.769	0.386^a^
	E3	Qizimei	30°02′	109°43′	1661	30	11.50	0.710	0.804	0.343^a^
Zhejiang (Z)	Z1	Qingliangfeng	30°06′	118°53′	975	25	6.25	0.535	0.621	0.167^a^
	Z2	Sihai	28°30′	120°43′	909	15	6.12	0.600	0.649	0.161
Taiwan (T)	T1	Chatian	24°47′	121°27′	1667	20	8.25	0.467	0.731	0.757^a^
	T2	Niaozui	24°44′	121°18′	1720	15	6.25	0.425	0.667	0.600^a^
	T3	Tong	24°31′	121°39′	1587	30	9.12	0.411	0.798	0.899^a^
Total	14					375	21.75	0.598	0.834	0.545^a^

a *P *<* *0.05.

The total DNA of dried leaves was extracted using the cetyltrimethyl ammonium bromide procedure (Doyle 1987) with some modifications and then stored at −20°C.

### PCR amplification, genotyping, and analysis of microsatellite markers of nDNA

A preliminary screening of microsatellite markers, or simple sequence repeat (SSR) markers, was performed using 40 SSR primers with 8% nondenaturing polyacrylamide gel electrophoresis for separation, as described by Tanaka et al. (1999), Asuka et al. (2004), and Ju et al. (2012). Linkage disequilibrium testing over all loci among populations was performed using FSTAT v2.9.3.2 (Goudet 1995) with the nominal level set at 0.05. Then, eight polymorphic SSRs with no linkage disequilibrium were selected: *mfc7* (Tanaka et al. 1999) and *sfc0007‐2*,* sfc0036*,* sfc0109*,* sfc0195‐2*,* sfc0378*,* sfc1063,* and *sfc1143* (Asuka et al. 2004). Amplification was carried out in a MyCycler Thermal Cycler (Bio‐Rad, Hercules, USA) under the following conditions: an initial 3 min at 94°C, followed by 36 cycles of 1 min at 94°C, 1 min at 55°C (*sfc0109*,* sfc0195‐2,* and *sfc1063*) or 58°C (*sfc0378* and *sfc1143*) or 60°C (*mfc7*,* sfc0007‐2,* and *sfc0036*) and 2 min at 72°C, with a final 10 min at 72°C. Amplification products were checked by capillary electrophoresis in an ABI 3730xl sequenator (ABI, Foster City, USA). Allele sizing was performed with GeneScan^™^ 500 LIZ^®^ size standard (ABI) and assigned with Allelogram v2.2 (http://code.google.com/p/allelogram).

Genetic variation was estimated by the average number of alleles (*A*) (Tajima 1983), observed and expected heterozygosity (*H*_O_ and *H*_E_, respectively) (Nei 1978) over all loci within and among populations, as well as at each locus among populations. The fixation index (FI) (Nei 1977) over all loci within and among populations was calculated. Among populations, FI and Hardy–Weinberg equilibrium tests (Guo and Thompson 1992) were carried out at each locus. In view of any departure from Hardy–Weinberg equilibrium probably due to the presence of null alleles (Soulsbury et al. 2007), the null allele frequency at each locus was calculated. As the presence of microsatellite null alleles was common in genetics studies, its effects could be moderate and negligible when its frequency was lower than 0.2, otherwise the large effects should be considered (Dakin and Avise 2004; Chapuis and Estoup 2007). Among populations, genetic differentiation was analyzed with the differentiation parameter (*R*_st_) (Michalakis and Excoffier 1996), and the isolation‐by‐distance (IBD) effect (Rousset 1997) was examined using the Mantel test (Mantel 1967; Smouse et al. 1986). These analyses here were all performed using Arlequin v3.1 (Excoffier et al. 2005), except the Hardy–Weinberg equilibrium tests and null allele frequency calculations with GENEPOP v4.0 (Rousset 2008).

In addition, Bayesian clustering analysis with a Markov Chain Monte Carlo (MCMC) approach over all loci was performed for individual clustering under a model assuming admixture and noncorrelated allele frequencies using Structure v2.1 (Pritchard et al. 2009). A series of 10 independent runs were performed for each of 1–15 clusters (*K*) with a burn‐in period of 10^5^ steps followed by 10^6^ replicates. The optimum was detected by calculating the parameter Δ*K* (Evanno et al. 2005), which considers the value of *K* corresponding to the maximum Δ*K* appropriate.

### PCR amplification, sequencing, and analysis of cpDNA sequence fragments

For amplification and sequencing of cpDNA, three polymorphic primers (3ʹ*trn*L–*trn*F, *trn*H–*psb*A, and *rpl*20–5ʹ*rps*12) were selected after a screening of 20 primers described by Taberlet et al. (1991), Hamilton (1999), and Shaw et al. (2007). Amplification reactions were performed in a volume of 20 *μ*L containing 10 *μ*L of 2× Taq PCR StarMix (GenStar, Beijing, China), 1 *μ*L each of forward and reverse primers (10 μmol/L), 1 *μ*L of template DNA (about 100 ng), and 7 *μ*L of ddH_2_O. Amplifications were carried out using the same PCR cycler as those for SSR amplification in section PCR amplification, genotyping and analysis of microsatellite markers of nDNA, with the following conditions: initial 3 min at 94°C, followed by 30 cycles of 1 min at 94°C, 1 min at 53°C and 2 min at 72°C, with a final 10 min at 72°C. Amplification products were checked by electrophoresis on 2% agarose gels stained with ethidium bromide and sequenced on a 3730xl DNA Analyzer (ABI).

DNA sequences were edited using Sequencher v5.0 (Gene Codes, Ann Arbor, USA) and aligned using Clustal X v1.81 (Thompson et al. 1997) with manual refining.

The distribution of haplotype frequency was mapped using ArcGIS 9.3 (http://www.esri.com). A statistical parsimony network of haplotypes was generated using TCS v1.21 with a median‐joining algorithm using a maximum‐parsimony (MP) approach (Clement et al. 2000). A Bayesian phylogenetic tree of haplotypes was constructed using MrBayes v3.2.0 (Ronquist et al. 2012), which employed a Bayesian MCMC approach run for 10^7^ iterations (burn‐in 1000) with parameters sampled every 1000 steps. Based on 1000 replicates, the bootstrap support values of the tree nodes resulting from maximum‐likelihood (ML) approaches with Akaike information criterion and Bayesian information criterion (BIC) procedures, with the best substitution models TPM3uf and F81, respectively, inferred by jModelTest 2 (Posada 2008; Darriba et al. 2012), then a MP approach and a neighbor‐joining (NJ) approach using PAUP v4.0b10 (Swofford 1998) were also presented. The corresponding sequences including 3ʹ*trn*L–*trn*F, *trn*H–*psb*A, and *rpl*20–5ʹ*rps*12 of “OutGroup” in the minimum spanning network and phylogenetic tree were extracted from *F. lucida*, which is the closest in this genus to *F. hayatae* (Ji et al. 2002).

Using Arlequin v3.1, genetic variation within and among populations of *F. hayatae* was estimated as haplotype diversity (*H*_d_) and average number of pairwise differences between haplotypes (*π*) (Nei and Li 1979; Tajima 1993), and analysis of genetic differences among populations was performed to infer genetic structure (Weir and Cockerham 1984; Excoffier et al. 1992). The differentiation parameter (*G*_st_) making use of haplotype frequencies was also used (Pons and Petit 1995). Thus, the relative rate of pollen and seed flow (*r*) could be estimated with FI over all loci among populations, *R*_st_ and *G*_st_ (Ennos 1994; Petit et al. 2005):

$$r=\left(\text{FI}+1\right)\times \frac{(1/R_{\mathrm{st}})-1}{(1/G_{\mathrm{st}})-1}-2$$

In addition, *N*_st_, a measure of genetic differentiation taking into account the distance between haplotypes, was estimated and compared with *G*_st_ using the program PermutandCpSSR v2.0 (Pons and Petit 1996; Burban et al. 1999). A significantly larger *N*_st_ value under *U*‐statistic test indicated a significant phylogeographic structure with closely related haplotypes found often in the same area, and that genetic distances increase with increasing geographic instances among populations (El Mousadik and Petit 1996).

Finally, Arlequin v3.1 was also used for Tajima's *D* (Tajima 1989) and Fu's *F*_s_ (Fu 1997) tests of neutrality among populations. Recent species range or demographic expansion was examined using a mismatch distribution analysis (Slatkin and Hudson 1991; Rogers and Harpending 1992; Schneider and Excoffier 1999).

## Results

### SSR‐based genetic variation within and among populations

The values of *A*,* H*_O_, *H*_E,_ and FI over all loci are listed in Table 1. *A* and FI for each population varied within 6.12–11.75 and 0.029–0.899, respectively, with high values of *A,* but also statistically significant FI values detected in populations in Taiwan (Table 1). The same significant FI and heterozygosity deficiency occurred in most populations and among all 14 populations (Table 1). At each locus, with relatively low null allele frequency (<0.2), high FI values with heterozygosity deficiency and significant departure from Hardy–Weinberg equilibrium among populations were detected (Table S1). Over eight loci, genetic differentiation with *R*_st_ = 0.233 was detected. The Mantel test applied for all 14 populations over eight loci did not show a significant IBD effect (*R* ^2^ = 0.0920, *P *=* *0.21) (Fig. S1).

### Bayesian clustering analysis

**Figure 2:**
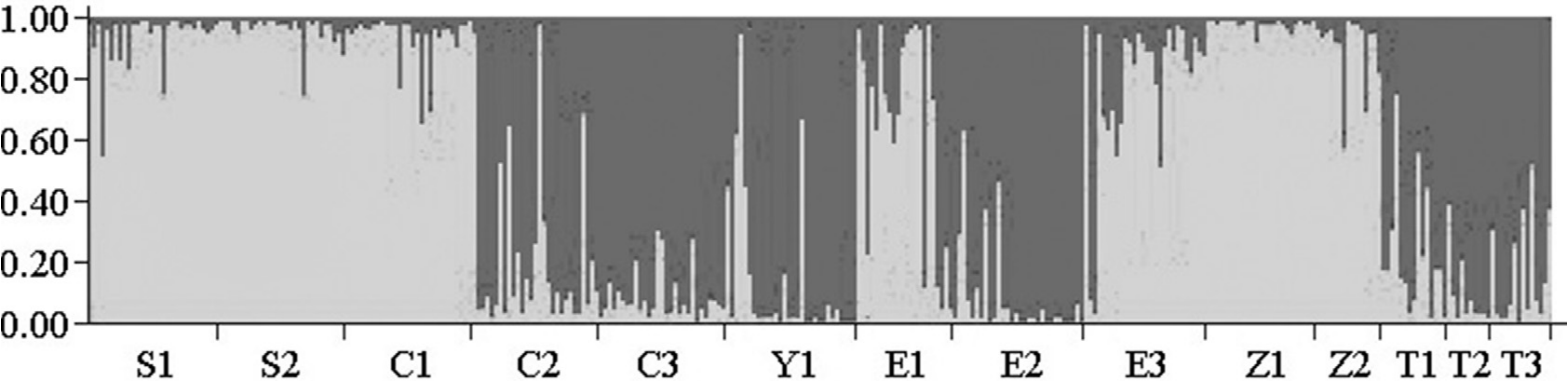
Bayesian clustering result with two distinct clusters in two colors.

Over eight loci, all 14 populations were most likely clustered into two groups, corresponding to the largest Δ*K* value (Fig. S2). Populations S1, S2, C1, E1, E3, Z1, and Z2 were clustered into one group and the others into another group, but there was mismatching between populations and genetic groups for some individuals (Fig. 2). For example, some individuals of population C2 were classified into genetic groups S1, S2, C1, E1, E3, Z1, and Z2.

### cpDNA haplotypes and their distributions

With a total of 1249 bp spliced, the aligned sequences of 3ʹ*trn*L–*trn*F, *trn*H–*psb*A, and *rpl*20–5ʹ*rps*12 from 14 populations of *F. hayatae* were 169, 343, and 737 bp, respectively, and a total of 22 variable sites, including 11 substitutions and 11 insertion/deletions, were detected in defining 15 haplotypes (Table S2). Haplotype H1 was fixed in the populations sampled in the Sichuan Basin, and the haplotypes fixed in the populations in Taiwan Island were more diverse. Additionally, a major specific haplotype was fixed with high frequency in most populations (Fig. 1A).

**Figure 3:**
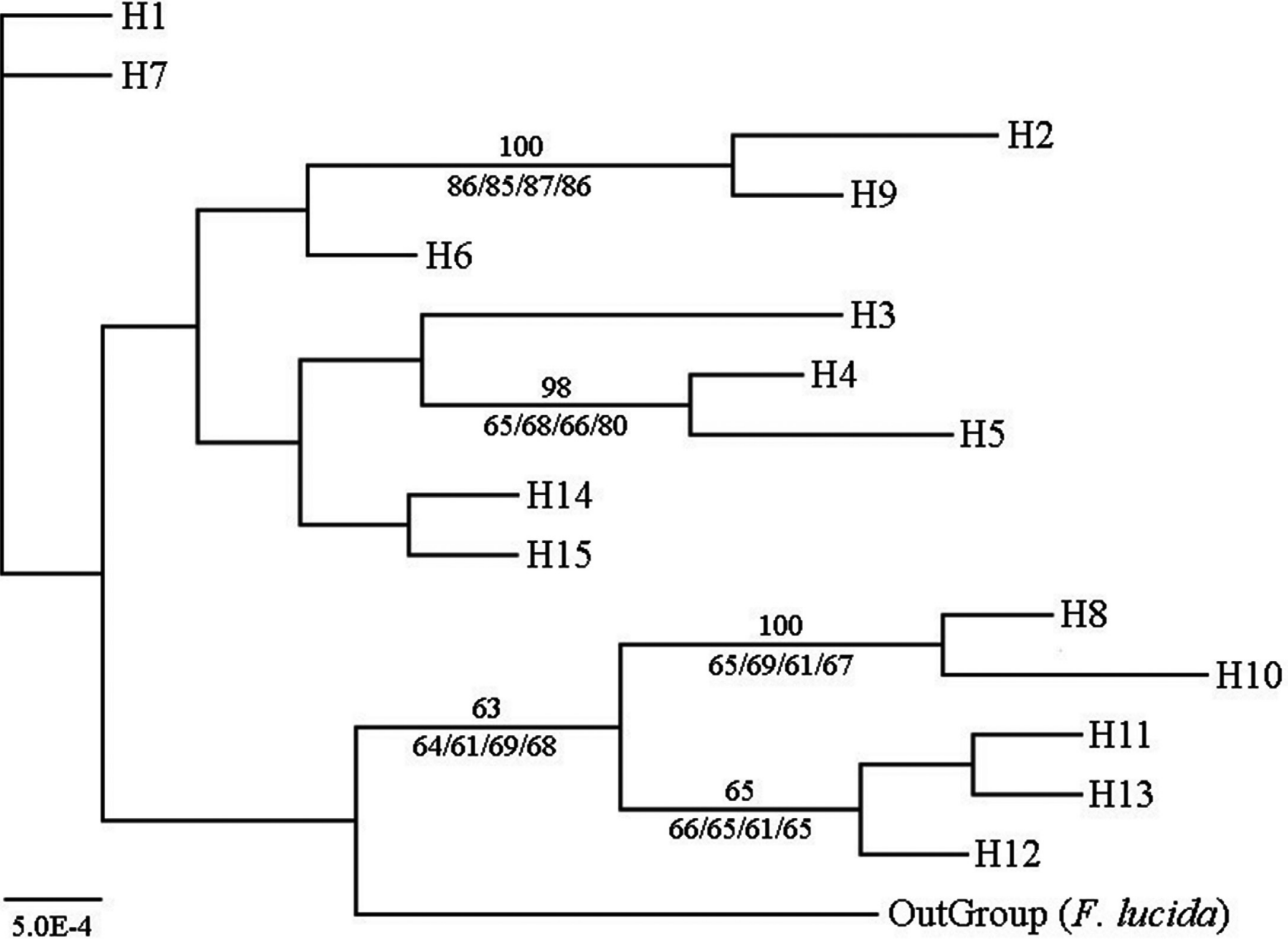
Bayesian phylogenetic tree of 15 haplotypes with “OutGroup” (*Fagus lucida*). Values above branches corresponding to posterior probabilities (%) and only those over 50% are shown. Values below branches correspond to bootstrap supports (%) of the nodes resulting from ML with Akaike information criterion, ML with Bayesian information criterion, MP and NJ phylogenetic inferences, and only those over 50% are shown.

The statistical parsimony network with “OutGroup” (*F. lucida*) indicated not only many interior nodes but also many missing intermediate haplotypes (Fig. 1B). In the Bayesian phylogenetic tree, the “OutGroup” branch was no older than that of H1 or H7, whereas its low posterior probability was detected (Fig. 3). In the other haplotypes recovered, H6 in population E3, H14, and H15 in populations Z1 and Z2 were older than others (Fig. 3). In addition, several haplotypes fixed in populations in Taiwan (H8 in T1; H10 in T2; and H11, H12 and H13 in T3) were clustered in a clade with two sub‐branches, while H4 and H5 were clustered into another clade, as shown by high posterior probabilities of Bayesian inference for these clades, which were also highly supported by the MP and NJ, as well as ML with two procedures (Fig. 3).

### Haplotype diversity and differentiation

*H*_d_ and *π* values varied within 0–0.385 and 0–7.86 × 10^−4^, respectively, with the first and second highest values found in Taiwan populations (Table 2). The among‐population values in the Sichuan Basin (*H*_d_ = 0.0342, *π *= 7.50 × 10^−5^) were lower than those outside (*H*_d_ = 0.834, *π *= 7.77 × 10^−3^); and values among all 14 populations (*H*_d_ = 0.714, *π *= 4.87 × 10^−3^) were higher than the mean (*H*_d_ = 0.0864, *π *= 1.45 × 10^−4^) (Table 2).

**Table 2:** *H*_d_ and *π* based on cpDNA sequence fragments within and among populations

	Population	*H*_d_	*π*
In Sichuan Basin	S1	0.0690	0.000170
	S2	0	0
	C1	0.0667	0.000163
	C2	0.0667	0.000109
	C3	0	0
	Y1	0	0
	Mean	0.0337	0.0000737
	Among populations	0.0342	0.0000750
Outside Sichuan Basin	E1	0.142	0.000116
	E2	0.133	0.000108
	E3	0	0
	Z1	0	0
	Z2	0.143	0.000234
	T1	0.385	0.000786
	T2	0	0
	T3	0.205	0.000345
	Mean	0.126	0.000199
	Among populations	0.834	0.00777
Over 14 populations	Mean	0.0864	0.000145
	Among populations	0.714	0.00487

The results showed significantly high genetic differentiation among all 14 populations with *G*_st_ = 0.712. Very high genetic differentiation was also detected for populations outside the Sichuan Basin (*G*_st_ = 0.829) but little for those inside. The relative rate of pollen over seed flows (*r*) was 10.6 among the 14 populations.

Phylogeographic structures were detected among all 14 populations, and populations outside the Sichuan Basin, with correspondingly significantly higher *N*_st_ values (0.977 and 0.980, respectively) than *G*_st_ values (0.712 and 0.829, respectively) (*P *<* *0.001). However, there was no significant difference between *N*_st_ (0.009) and *G*_st_ (0.005) among populations in the Sichuan Basin (*P *>* *0.1).

### Test of neutrality and mismatch distribution

The results of Tajima's *D* and Fu's *F*_s_ tests of neutrality were not significant among all 14 populations and for populations outside the Sichuan Basin, while there were conflicting results between the two tests among populations within the Sichuan Basin (Table S3). However, all the mismatch distributions were roughly multimodal (Fig. S3), suggesting no signals of population expansion in the analysis.

## Discussion

### Genetic diversity

Based on SSR of nDNA, the average number of alleles (*A*) was 21.75 among the 14 *F. hayatae* populations (Table 1), which was much higher than for any other *Fagus* species studied, such as *F. longipetiolata* (*A *=* *4.9, Liu 2008), *Fagus crenata* (*A *=* *9.9, Tanaka et al. 1999), *Fagus japonica* (*A *=* *8.64, Hiraoka and Tomaru 2009a), *Fagus sylvatica* (*A *=* *10.75, Vornam et al. 2004), *Fagus orientalis* (*A *=* *10.33, Shanjani et al. 2010), and *Fagus grandifolia* (*A *=* *13.8, Koch et al. 2009). Our study was in accord with the results of Kung (2012), which also showed an average of 24.33 alleles for populations mainly from Taiwan.

By sequencing 1249‐bp cpDNA fragments, we detected 15 haplotypes over 345 individuals in 14 *F. hayatae* populations. This was generally more than that detected in other *Fagus* species using the same analysis, such as *F. lucida* (14 haplotypes, 1918 bp, 276 individuals, and 21 populations, Zhang et al. 2013), *F. longipetiolata* (13 haplotypes, 1664 bp, 201 individuals, and 26 populations, Liu 2008; and 14 haplotypes, 1918 bp, 276 individuals, and 28 populations, Zhang et al. 2013), *F. engleriana* (six haplotypes, 2149 bp, 350 individuals, and 25 populations, Lei et al. 2012), *F. crenata* (13 haplotypes, 3229 bp, 109 individuals, and 45 populations, Fujii et al. 2002; and seven haplotypes, 1515 bp, 351 individuals, and 21 populations, Okaura and Harada 2002), and *F. grandifolia* (20 haplotypes, 1920 bp, 232 individuals, and 137 populations, Morris et al. 2010). Studies of *F. sylvatica* with chloroplast cleaved amplification polymorphism sequence‐tagged sites detected 11 haplotypes from 399 individuals in 85 populations (Demesure et al. 1996) and 14 haplotypes from 335 individuals in 67 populations (Vettori et al. 2004). A similar study of *F. orientalis* with chloroplast microsatellites detected eight haplotypes from 562 individuals in 13 populations (Shanjani et al. 2010).

Ellegren (2009) suggested that high genetic diversity generally resulted from large populations and high mutation rates, while the latter may make more contribution. Considering the very sparsely scattered distributions of *F. hayatae* (Zhang et al. 2013; Shen et al. 2015) populations across the rather wide range, genetic mutation in the mutually isolated habitats might play a primary role in forming the relatively high genetic diversity of *F. hayatae*, compared with the *Fagus* species mentioned above. Zhang et al. (2013) suggested that generally low numbers of missing intermediate haplotypes in the statistical parsimony network indicated low extinction rates. In contrast, many missing intermediate haplotypes in this study (Fig. 1B) may also be related to the relatively high mutation rate of *F. hayatae*. Thus, the lower genetic diversity of populations in the Sichuan Basin compared to that outside (Table 2) indicated that the corresponding mutation rate in the Sichuan Basin was relatively low.

### Genetic structure

Differences between biparentally inherited nDNA SSR markers and maternally inherited cpDNA sequence fragments have been used to detect genetic differentiation among populations (Petit et al. 2005; Cornman and Arnold 2007), and it is important to combine the two approaches to reveal genetic structure and the historical reasons underlying it (Hodgins and Barrett 2007; Bai et al. 2010).

Over eight SSR loci, our results indicated inbreeding in *F. hayatae* among the 14 populations, and significant FI with heterozygosity deficiency in most populations (Table 1). It is suggested that inbreeding in this wind‐pollinated species is mainly due to habitat fragmentation with limited pollen flow (Knapp et al. 2001; Jump and Peñuelas 2006). Thus, with little influence of null alleles, significant FI and departures from the Hardy–Weinberg equilibrium among populations of *F. hayatae* at each locus (Table S1) must also result from isolation and fragmentation by mountains in subtropical China. It is noteworthy that *F. hayatae* had *R*_st_ = 0.233, comparable to other *Fagus* species distributed in China (e.g., *F. longipetiolata*,* R*_st_ = 0.232, Liu 2008; and *F. engleriana*,* R*_st_ = 0.263, Zhou and Fang 2012), but much higher than those *Fagus* species distributed in other regions (e.g., *F. japonica*,* R*_st_ = 0.025, Hiraoka and Tomaru 2009a; *F. crenata*,* R*_st_ = 0.041, Hiraoka and Tomaru 2009b; *F. sylvatica*,* R*_st_ = 0.054, Comps et al. 1990; *F. orientalis*,* R*_st_ = 0.048, Shanjani et al. 2010; and *F. grandifolia*,* R*_st_ = 0.168, Kitamura and Kawano 2001) and that of the mean of 77 angiosperm species (*R*_st_ = 0.184, Petit et al. 2005).

Based on SSR analysis, the Mantel test showed no significant IBD effect among populations over eight loci (Fig. S1). Two clusters each with mixed populations in different locations and many mismatched individuals also indicated that genetic distance was not correlated with geographic distance (Fig. 2). It was that broadly and randomly occurring mutation of nDNA played an important role in shaping population genetic structure, as SSR genetic mutation could be of relatively great historical importance for the lack of IBD effect (Hutchison and Templeton 1999).

The chloroplast genetic differentiation of *F. hayatae* (*G*_st_ = 0.712), as well as of other *Fagus* species (*F. lucida*,* G*_st_ = 0.705, Zhang et al. 2013; *F. longipetiolata*,* G*_st_ = 0.900 and 0.936, Liu 2008 and Zhang et al. 2013; *F. engleriana*,* G*_st_ = 0.831, Lei et al. 2012; *F. sylvatica*,* G*_st_ = 0.902, Demesure et al. 1996; and *F. orientalis*,* G*_st_ = 0.800, Shanjani et al. 2010), was also higher than that of the mean for 124 angiosperm species (*G*_st_ = 0.637, Petit et al. 2005). The low seed flow mainly responsible for the high differentiation detected in other *Fagus* species (Tomaru et al. 1998; Kitamura and Kawano 2001; Rowden et al. 2004; Liu 2008; Lei et al. 2012; Zhang et al. 2013) may also be the case for *F. hayatae*. Therefore, the inefficient seed dispersal strategy with predation of vertebrates and habitat fragmentation may have jointly resulted in the genetic isolation among populations.

In addition, Petit et al. (2005) showed that the median of *r* is about 17 in seed plants, suggesting a general asymmetry between gene flows, as gene exchange through pollen is much more important than through seeds. However, the value of *r = *10.6 in *F. hayatae* was much lower compared with other *Fagus* species (e.g., *F. longipetiolata*,* r *=* *34.2, Liu 2008; *F. sylvatica*,* r *=* *84, Demesure et al. 1996; and *F. orientalis*,* r *=* *44, Shanjani et al. 2004), suggesting a stronger genetic isolation with also a limitation to pollen flow, possibly due to the more spatially isolated distribution compared to other *Fagus* species in China (Shen et al. 2015).

The results of tests between *N*_st_ and *G*_st_ showed that significant phylogeographic structure among all 14 populations was mainly from the contribution of high genetic differentiation among populations outside the Sichuan Basin. In contrast, within the Sichuan Basin, no significant phylogeographic structure (with *G*_st_ close to 0 and almost 100% variation within populations) suggested that these populations may have had a single origin and long existed within an enclosed environment, that is the Sichuan Basin.

### Demographic history and glacial refugia

The phylogenetic tree showed that H1 and H7 were older than the other haplotypes (Fig. 3). The fact that it is very common for beech species to share haplotypes (Zhang et al. 2013) indicates that H1 and H7 may be shared by *F. hayatae* and other *Fagus* species and that population T1 and populations in the Sichuan Basin were more ancient elements of *F. hayatae*. Zhang et al. (2013) also suggested that Chinese beeches started to diversify on entering the Pliocene and that this diversification intensified during the Pleistocene, thus more haplotypes in the phylogenetic tree (Fig. 3) were young and branched during the Quaternary. The lack of evidence for population expansion, according to the tests of neutrality and mismatch distribution (Table S3, Fig. S3), suggested that *F. hayatae* may have experienced a long isolation with limited admixture and independent evolution among populations.

The genetic structure in *F. hayatae* could be due to its demographic dynamics during the Quaternary. Pollen records indicate that the ancestor of *F. hayatae* migrated to southern China possibly from continental northeast Asia during the Pliocene (Denk and Grimm 2009), suggesting that the species was probably widespread in north China during the Tertiary. Migration of *F. hayatae* southwards to mountainous areas of subtropical China may have occurred in the early Pleistocene when the climate became colder (Liu et al. 2003). Possible migration and admixture among populations of the ancestors of *F. hayatae* could also have occurred (Denk and Grimm 2009), especially for populations with close locations, such as E1 and E2 in Hubei with haplotypes H4 and H5 clustered in one clade in the phylogenetic tree (Fig. 3). After the admixture disappeared, outside the Sichuan Basin the populations evolved independently with high mutation rate (see section Genetic diversity). In the Sichuan Basin, however, relatively low mutation rate (see section Genetic diversity) and the decline in gene flow or exchange with outside populations allowed the single ancient haplotype H1 to spread widely among populations in this enclosed environment.

Thus, the existence of multiple refugia for *F. hayatae* in mountains of subtropical China during the glaciations was critical for the contemporary genetic spatial structure of the species. Mountains around the Sichuan Basin could be glacial refugia of *F. hayatae*, with the fixed haplotype H1 firstly derived from the ancestor. To‐and‐fro migrations in *F. hayatae* between the Sichuan Basin and surrounding mountains occurred during the glacial and interglacial periods. However, the range of its distribution and migration gradually reduced and was circumscribed to low‐altitude areas close to mountains, and the species rarely entering lowlands during the Quaternary due to moisture limitation (Liu et al. 2003). This was also a critical factor that drove the upward migration in mountains surrounding the Sichuan Basin rather than northward expansion, facing climate warming after the last glacial period (Cao et al. 1995; Fang and Lechowicz 2006; Shen et al. 2015).

Mountains and hills in southeast China, including Taiwan Island, could also be glacial refugia where abundant haplotypes were fixed. Similar to the case in the Sichuan Basin, the distribution range of *F. hayatae* gradually reduced in each isolated mountain during the Pleistocene, consistent with the pollen evidence indicating that it was either more extensive or lower in altitude than it is today (Zheng and Li 2000). The isolated pattern was maintained or even enhanced by the monsoon climate in the postglacial period (Fang and Lechowicz 2006; Guo and Werger 2010; Shen et al. 2015). The fact that *F. hayatae* is now absent from the Dabie or Luoxiao Mountains located at the same latitude and between the sampled populations in Hubei and Zhejiang, respectively, is an unresolved issue, requiring further information from pollen and perhaps also field exploration in these two mountains.

Hewitt (2000) suggested that during the Quaternary, climate change and the varied topography in southern temperate regions and the tropics tended to isolate species into populations that evolved independently with only occasional gene flow, as confirmed for *F. hayatae* in subtropical China. Postglacial expansion has not been detected in any *Fagus* species studied in China (Liu 2008; Lei et al. 2012; Zhang et al. 2013), including *F. hayatae* in the present study, in contrast to the rapid northward range expansion for *Fagus* species in Japan (Okaura and Harada 2002; Hiraoka and Tomaru 2009b), Europe (Demesure et al. 1996; Magri et al. 2006), and North America (Kitamura and Kawano 2001). The potential environmental or ecological barriers may have acted as refugia for *Fagus* in the Quaternary Ice Ages.

### Conservation implications

Our results could provide information useful for the conservation of not only *F. hayatae*, a species on the China Species Red List, but also other endemic (especially paleo‐endemic) plant species assembled in the mountains of subtropical China. Ying (2001) localized three hot spots of plant diversity and endemism in China: the Hengduan Mountain Ranges, the Central China region, and the Southern Mountain Ranges. The populations of *F. hayatae* in Sichuan and Hubei were located in the Central China region and may be better protected than those in Zhejiang and Taiwan which are on the east border of the species range. The populations Z1 and Z2 in Zhejiang were small, with nearly only a single fixed haplotype, and so should be given more attention. Despite the high haplotype diversity of *F. hayatae* in Taiwan, isolation with inbreeding could leave populations at risk. Although a high genetic diversity remains among all populations of *F. hayatae*, genetic isolation with high genetic differentiation resulting from habitat fragmentation caused by natural and anthropogenic factors needs to be taken into account in conservation of this relict species.

## Conclusions

Molecular evidence – nDNA SSR loci and cpDNA sequence fragments – showed that *F. hayatae*, the *Fagus* species endemic to China with the most dispersed distribution, had a demographic history with multiple refugia during glacial periods and an isolated population structure with high genetic differentiation, in which climate change during the Quaternary and genetic mutation may have played important roles. Despite some differences between the populations in and outside the Sichuan Basin, long‐term genetic isolation among populations with habitat fragmentation was the main characteristic of *F. hayatae*.

## Conflict of Interest

None declared.

## Supporting information


**Table S1.** Genetic diversity, FI, *P‐*values of Hardy–Weinberg equilibrium tests and null allele frequency at each SSR locus among populations of *F. hayatae*.
**Table S2.** Variable sites of 1249‐bp spliced cpDNA sequence fragments in 15 haplotypes in 14 populations of *F. hayatae*.
**Table S3.** Results of neutrality tests.
**Figure S1.** Mantel tests based on nDNA SSR loci between genetic differentiation (*F*_st_) and geographical distance (*Dist*).
**Figure S2.** Relationship of Δ*K* with the number of clusters (*K*).
**Figure S3.** Mismatch distributions for populations (a) inside and (b) outside the Sichuan Basin, and (c) over the 14 populations, with black lines representing the distributions expected for expanding populations and red lines representing the observed mismatch distance.
