# Supplementary material for: The phylogeography of Fagus hayatae (Fagaceae): genetic isolation among populations
Source: Ecol Evol. 2016 Mar 21;6(9):2805–16. doi: 10.1002/ece3.2042 (PMC4801808; doi:10.1002/ece3.2042)

**Supporting Information**

**Table S1** Genetic diversity, *FI*, *P-*values of Hardy–Weinberg equilibrium tests and null allele frequency at each SSR locus among populations of *F. hayatae*.

**Table S2** Variable sites of 1249-bp spliced cpDNA sequence fragments in 15 haplotypes in 14 populations of *F. hayatae*.

**Table S3** Results of neutrality tests.

**Fig. S1** Mantel tests based on nDNA SSR loci between genetic differentiation (*F_st_*) and geographical distance (*Dist*).

**Fig. S2** Relationship of Δ*K* with the number of clusters (*K*).

**Fig. S3** Mismatch distributions for populations (a) inside and (b) outside the Sichuan Basin, and (c) over the 14 populations, with black lines representing the distributions expected for expanding populations and red lines representing the observed mismatch distance.

**Table S1**

| Locus | *H_O_* | *H_E_* | *FI* | *P*-value of Hardy–Weinberg equilibrium test | Null allele frequency |
| --- | --- | --- | --- | --- | --- |
| *mfc7* | 0.770 | 0.931 | 0.442* | < 0.001 | 0.082 |
| *sfc0007-2* | 0.412 | 0.655 | 0.351* | < 0.001 | 0.147 |
| *sfc0036* | 0.639 | 0.853 | 0.388* | < 0.001 | 0.117 |
| *sfc0109* | 0.770 | 0.956 | 0.663* | < 0.001 | 0.101 |
| *sfc0195-2* | 0.128 | 0.629 | 0.733 * | < 0.001 | 0.151 |
| *sfc0378* | 0.690 | 0.915 | 0.694* | < 0.001 | 0.131 |
| *sfc1063* | 0.576 | 0.829 | 0.523* | < 0.001 | 0.139 |
| *sfc1143* | 0.800 | 0.901 | 0.387* | < 0.001 | 0.059 |

* *P* < 0.05.

**Table S2**

| Total | | 171 | 2 | 1 | 52 | 4 | 29 | 11 | 1 | 2 | 15 | 17 | 1 | 1 | 25 | 13 | 345 |
| --- | --- | --- | --- | --- | --- | --- | --- | --- | --- | --- | --- | --- | --- | --- | --- | --- | --- |
| Sample sizes sequenced successfully | T3 |  |  |  |  |  |  |  |  |  |  | 17 | 1 | 1 |  |  | 19 |
|  | T2 |  |  |  |  |  |  |  |  |  | 15 |  |  |  |  |  | 15 |
|  | T1 |  |  |  |  |  |  | 11 | 1 | 2 |  |  |  |  |  |  | 14 |
|  | Z2 |  |  |  |  |  |  |  |  |  |  |  |  |  | 1 | 13 | 14 |
|  | Z1 |  |  |  |  |  |  |  |  |  |  |  |  |  | 24 |  | 24 |
|  | E3 |  |  |  |  |  | 29 |  |  |  |  |  |  |  |  |  | 29 |
|  | E2 |  |  |  | 27 | 2 |  |  |  |  |  |  |  |  |  |  | 29 |
|  | E1 |  |  |  | 25 | 2 |  |  |  |  |  |  |  |  |  |  | 27 |
|  | Y1 | 25 |  |  |  |  |  |  |  |  |  |  |  |  |  |  | 25 |
|  | C3 | 30 |  |  |  |  |  |  |  |  |  |  |  |  |  |  | 30 |
|  | C2 | 29 |  | 1 |  |  |  |  |  |  |  |  |  |  |  |  | 30 |
|  | C1 | 29 | 1 |  |  |  |  |  |  |  |  |  |  |  |  |  | 30 |
|  | S2 | 30 |  |  |  |  |  |  |  |  |  |  |  |  |  |  | 30 |
|  | S1 | 28 | 1 |  |  |  |  |  |  |  |  |  |  |  |  |  | 29 |
| *rpl*20-5’*rps*12 (513-1249bp) | 1215-1219 | - - - - - | - - - - - | - - - - - | ACTTT | ACTTT | ACTTT | - - - - - | - - - - - | - - - - - | - - - - - | - - - - - | - - - - - | - - - - - | - - - - - | - - - - - |  |
|  | 1169 | A | A | A | A | A | A | A | A | A | A | G | G | G | A | A |  |
|  | 1100 | A | A | A | A | A | A | A | T | A | T | A | A | A | A | A |  |
|  | 1021 | C | T | C | C | C | C | C | C | T | C | C | C | C | C | C |  |
|  | 1008 | G | A | G | G | G | G | G | G | G | G | G | G | G | G | G |  |
|  | 944 | A | T | A | A | A | A | A | A | T | A | A | A | A | A | A |  |
|  | 749 | T | T | T | T | T | T | T | G | T | G | G | G | G | T | T |  |
|  | 736 | - | - | - | - | - | - | - | - | - | - | A | A | A | - | - |  |
|  | 713 | G | G | G | A | A | G | G | G | G | G | G | G | G | G | G |  |
| *trn*H-*psb*A (170-512bp) | 469 | T | T | T | T | T | T | T | T | T | G | T | T | T | T | T |  |
|  | 453 | T | T | T | T | T | - | - | - | - | - | T | - | T | T | T |  |
|  | 408 | - | - | - | A | A | - | - | - | - | - | - | - | - | - | - |  |
|  | 407 | - | - | - | A | A | A | - | - | - | - | - | - | - | A | - |  |
|  | 406 | A | A | A | A | A | A | - | - | - | - | - | - | - | A | - |  |
|  | 397 | T | T | A | T | T | T | T | T | T | T | T | T | T | T | T |  |
|  | 375 | A | A | G | A | A | A | A | A | A | A | A | A | A | A | A |  |
|  | 366-368 | - - - | - - - | - - - | - - - | - - - | - - - | - - - | - - - | - - - | - - - | TTT | TTT | - - - | - - - | - - - |  |
|  | 343 | - | - | - | - | - | - | - | - | - | T | - | - | - | - | - |  |
|  | 231-245 | - - - - - - - - - - - - - - - | - - - - - - - - - - - - - - - | - - - - - - - - - - - - - - - | - - - - - - - - - - - - - - - | - - - - - - - - - - - - - - - | - - - - - - - - - - - - - - - | - - - - - - - - - - - - - - - | - - - - - - - - - - - - - - - | - - - - - - - - - - - - - - - | TTATTTAATTACTAT | - - - - - - - - - - - - - - - | - - - - - - - - - - - - - - - | - - - - - - - - - - - - - - - | - - - - - - - - - - - - - - - | - - - - - - - - - - - - - - - |  |
|  | 220 | - | - | - | A | A | - | - | - | - | - | - | - | - | - | - |  |
| 3’*trn*L-*trn*F (1-169bp) | 104 | T | T | T | T | T | T | T | - | T | - | T | T | T | T | T |  |
|  | 97 | C | C | C | C | T | C | C | C | C | C | C | C | C | C | C |  |
| Haplotype | | H1 | H2 | H3 | H4 | H5 | H6 | H7 | H8 | H9 | H10 | H11 | H12 | H13 | H14 | H15 | Total |

**Table S3**

|  | Tajima’s *D* | Fu’s *F_s_* |
| --- | --- | --- |
| In Sichuan Basin | –1.75 * | –2.29 |
| Outside Sichuan Basin | –0.0101 | 12.2 |
| Over 14 populations | –1.17 | 5.47 |

* *P* < 0.05.

**Fig. S1**


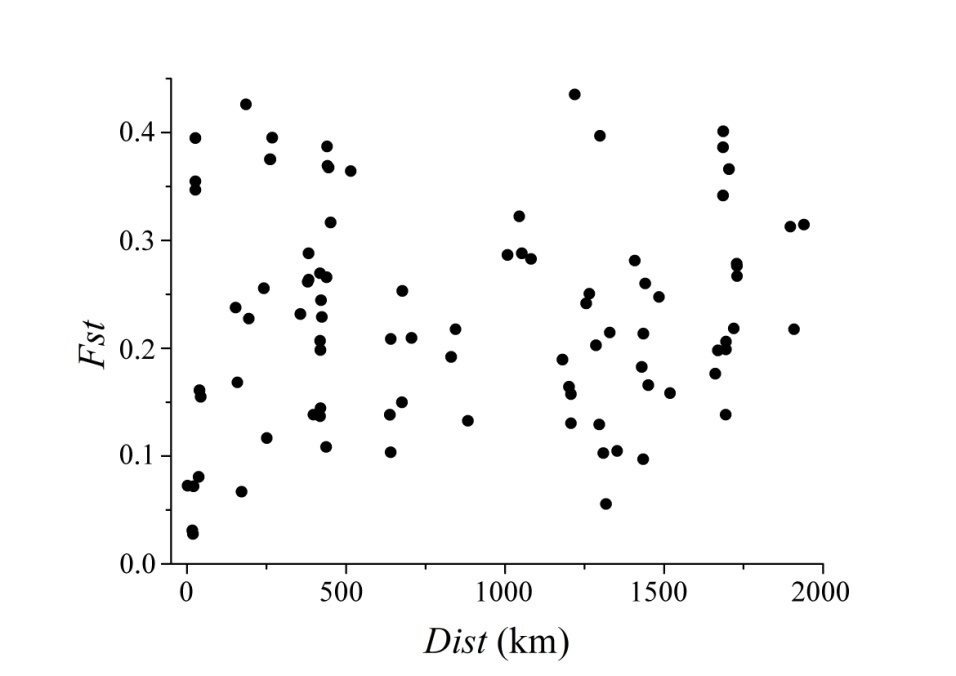


**Fig. S2**


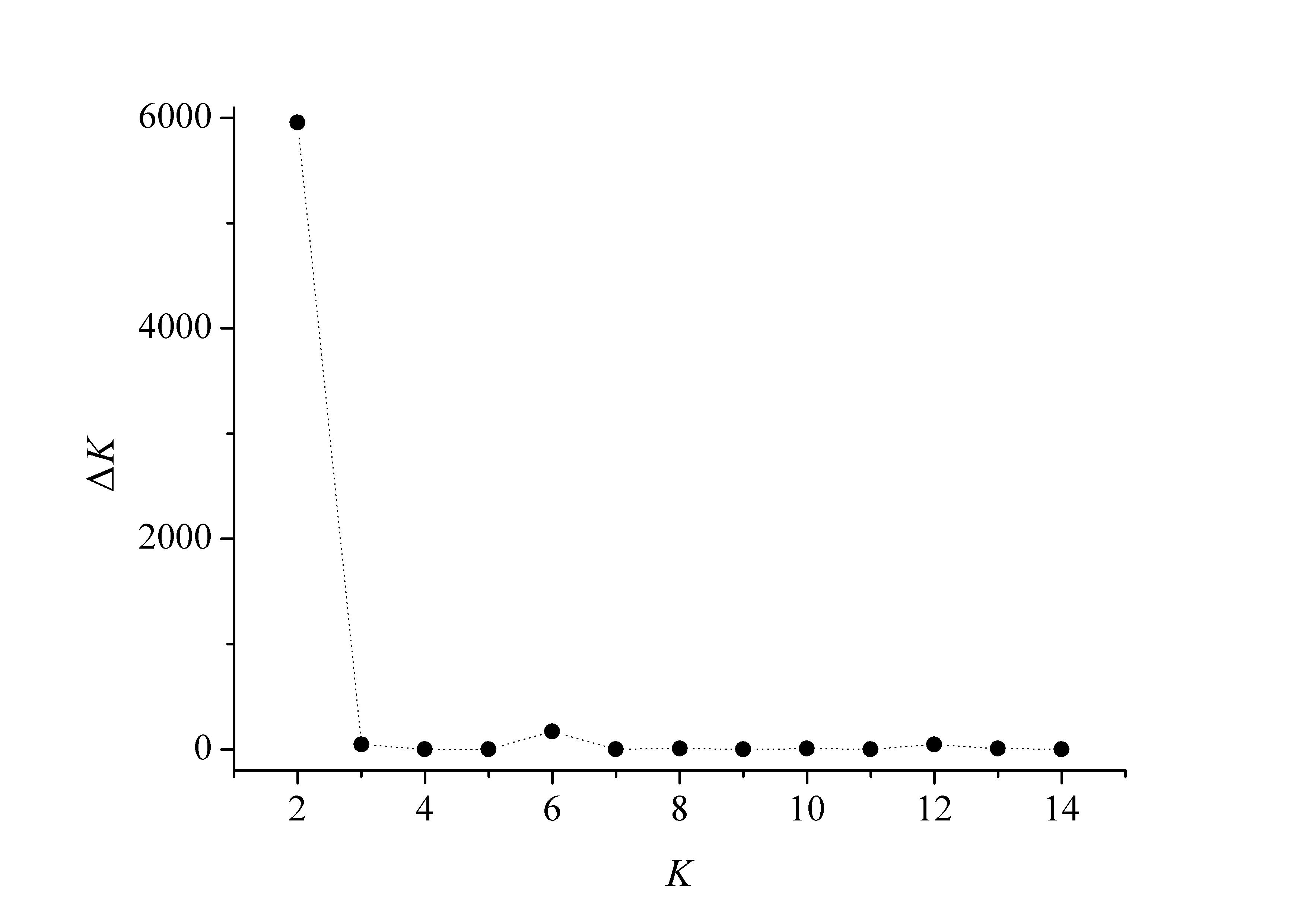


**Fig. S3**


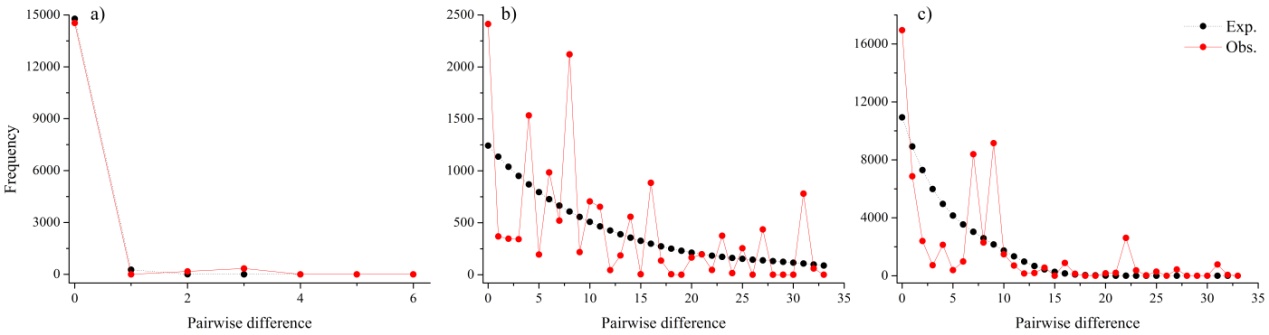

Supplement: Supplementary file 1 — Table S1. Genetic diversity, FI, P‐values of Hardy–Weinberg equilibrium tests and null allele frequency at each SSR locus among populations of F. hayatae. Table S2. Variable sites of 1249‐bp spliced cpDNA sequence fragments in 15 haplotypes in 14 populations of F. hayatae. Table S3. Results of neutrality tests. Figure S1. Mantel tests based on nDNA SSR loci between genetic differentiation (F st) and geographical distance (Dist). Figure S2. Relationship of ΔK with the number of clusters (K). Figure S3. Mismatch distributions for populations (a) inside and (b) outside the Sichuan Basin, and (c) over the 14 populations, with black lines representing the distributions expected for expanding populations and red lines representing the observed mismatch distance. [file ECE3-6-2805-s001.docx]
